# Beyond the Lockdown Binary: Family Caregiver Needs for Creative Solutions During a Global Pandemic

**DOI:** 10.1093/geroni/igab046.2114

**Published:** 2021-12-17

**Authors:** Gwen McGhan, Kristin Flemons, Deirdre McCaughey, Whitney Hindmarch

**Affiliations:** University of Calgary, Calgary, Alberta, Canada

## Abstract

As COVID-19 lockdowns began in Canada last spring, family caregivers (FCGs) of people living with dementia (PLWD) found themselves facing a catch-22: they and their family members were often most at risk of severe outcomes should they contract the virus, yet the public health measures put in place also detrimentally affected their ability to continue providing care. To understand the nuances of caregiver experiences during the pandemic, we conducted 9 focus groups with 19 FCGs of PLWD in the Calgary region in summer 2020. Caregivers reported negative outcomes resulting from decreased services for both themselves and the PLWD, including increased isolation, poor mental health, and accelerated dementia progression. Caregivers also emphasized the importance thinking beyond the binary of either locking down or opening up; rather, we must find creative solutions to safely continue providing supports to caregivers. This presentation explores FCG suggestions for balancing COVID-19 risk against caregiver needs.

